# A role for pathogenic autoantibodies in small fiber neuropathy?

**DOI:** 10.3389/fnmol.2023.1254854

**Published:** 2023-09-20

**Authors:** Omar Daifallah, Adham Farah, John M. Dawes

**Affiliations:** ^1^Department of Zoology, King Saud University, Riyadh, Saudi Arabia; ^2^Nuffield Department of Clinical Neurosciences, University of Oxford, Oxford, United Kingdom

**Keywords:** autoantibodies, small fiber neuropathy (SFN), neuropathic pain (NeP), intravenous immunoglobulin (IVIG), passive transfer models, novel autoantibodies

## Abstract

The immune system has a role in neuropathic pain which includes autoimmune mechanisms (e.g., autoantibodies). Clinical studies have identified a number of conditions where neuropathic pain is common and that are associated with autoantibodies targeting antigens within the nervous system. Interestingly sensory symptoms can be relieved with immunotherapies or plasma exchange, suggesting that pain in these patients is antibody-mediated. Recent preclinical studies have directly addressed this. For example, passive transfer of CASPR2 autoantibodies from patients cause increased pain sensitivity and enhanced sensory neuron excitability in mice confirming pathogenicity and demonstrating that patient autoantibodies are a mechanism to cause neuropathic pain. Small fiber neuropathy (SFN) exclusively affects small sensory fibers (typically nociceptors) and is characterized by severe neuropathic pain. Known causes include diabetes, B12 deficiency and rare variants in sodium channel genes, although around 50% of cases are idiopathic. SFN is associated with autoimmune conditions such as Sjorgen’s syndrome, Sarcoidosis and Celiac disease and immunotherapy in the form of Intravenous immunoglobulin (IVIG) has proved an effective treatment. Autoantibodies have been identified and, in some cases, passive transfer of SFN patient IgG in mice can recapitulate neuropathic pain-like behavior. Here we will discuss clinical and preclinical data relating to the idea that pathogenic autoantibodies contribute to SNF. We discuss putative pathogenic antibodies, cellular targets and the molecular mechanisms by which they cause sensory neuron damage and the development of neuropathic pain. Finally, we will comment on future directions which may provide further insights into the mechanisms underlying SFN in patients.

## 1. Introduction

Neuropathic pain results from damage or disease of the somatosensory system. With its negative impact on quality of life and high prevalence within the population (7–10%) it presents a heavy burden worldwide despite advances in diagnostic tools and treatments (Colloca et al., 2017). One of its most cryptogenic etiologies is Small Fiber Neuropathy (SFN), a peripheral nerve condition where preferential damage occurs to thinly myelinated Aδ and unmyelinated C fibers. This results in severe pain which typically, but not always, manifests in a length manner, and can be coupled with autonomic dysfunction (Gemignani et al., 2022). Adequate studies of SFN prevalence are limited, with estimates ranging from 50 to 130 cases per 100,000 of the population (Peters et al., 2013; Bitzi et al., 2021). There is a consensus that SFN is likely underdiagnosed, for example, its presence in fibromyalgia (FMS) patients suggests a much higher global prevalence (Oaklander and Nolano, 2019). Furthermore, with the presence of SFN in globally increasing diseases such as Diabetes mellitus, its prevalence is expected to rise in the coming years (Themistocleous et al., 2014).

**FIGURE 1:**
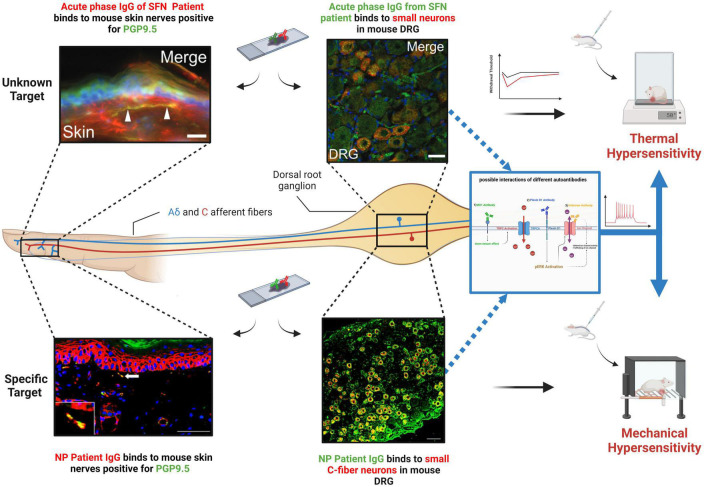
Binding of SFN patient IgG to small primary sensory neurons both in the skin and at the level of the DRG for both an unknown antigen **(top)** and a known target (Plexin-D1) **(bottom)**. When passively transferred to mice, this binding results in pain hypersensitivity. **(Top left Corner)** Adopted with permission from Yuki et al. (2018), Scale bar = 20 μm. **(Top Middle)** Adopted with permission from Yuki et al. (2018), Scale bar = 10 μm. (Bottom left Corner) Patient IgG containing Plexin-D1 autoantibodies. Adopted with permission from Fujii et al. (2018) Scale bar = 50 μm. **(Bottom Middle)** Patient IgG containing Plexin-D1 autoantibodies. Adopted with permission from Fujii et al. (2018), Scale bar = 50 μm. **(Right middle diagram)** A schematic of possible effects of autoantibodies binding to antigens on sensory neurons. (1) Potential TRPC6 activation via MX1 antibodies. (2) pERK activation by Plexin-D1 binding might lead to increased ion channel activity. (3) Unknown autoantibody could bind directly to ion channels. NP, neuropathic pain. Created with BioRender.com.

Small fiber neuropathy patients can present with either somatic (e.g., pain, numbness) or autonomic (e.g., abnormal sweating, fatigue) symptoms, or both. SFN can be length-dependent, which follows a stocking-glove pattern, and is seen mostly in cases with metabolic disturbances, and non-length-dependent that manifests in a diffuse, patchy and asymmetrical manner and is predominantly seen in immune-related cases (Devigili et al., 2020). In diagnosis, normal nerve conduction studies are used to rule out large fiber dysfunction, and confirmation by intraepidermal nerve fiber density (IENFD) along with Quantitative Sensory Testing (QST) are considered the gold standard diagnostic tools (Devigili et al., 2020). Secondary SFN (sSFN) can occur due to genetic mutations in sodium channels, vitamin B12 deficiency, glucose intolerance/Diabetes as well as incidents occurring post-vaccination (Souayah et al., 2009; Eijkenboom et al., 2019; Waheed et al., 2021). However, around 50% of cases are idiopathic (iSFN) (de Greef et al., 2018). Dysfunction of the immune system is thought to contribute with around 20% of SFN cases being associated with autoimmune conditions (e.g., Sjorgen’s syndrome, sarcoidosis) (de Greef et al., 2018). Autoimmunity includes the action of autoantibodies and autoantibodies targeting antigens within the nervous system are known to have a role in other peripheral neuropathies and contribute to disease progression (Querol et al., 2023). Furthermore, studies have shown that autoantibodies can directly drive neuropathic pain in patients by targeting antigens on sensory neurons (Dawes et al., 2018), proposing similar mechanisms in SFN. In line with this, Intravenous immunoglobulin (IVIG) and plasma exchange, which can either block or remove circulating antibodies, can alleviate pain and other symptoms in SFN patients (Finsterer and Scorza, 2022), suggesting the existence of pathogenic autoantibodies. In agreement, autoantibodies have been identified in SFN (Pestronk et al., 2003; Antoine et al., 2015; Chan et al., 2022; see Table 1) which can bind small sensory neurons (Figure 1) and in some cases, pathogenicity has been confirmed with the use of passive transfer models (Yuki et al., 2018; Fujii et al., 2021).

**TABLE 1:** Immunotherapy studies of SFN associated with known autoantibodies.

Target(s)	Number of participants	Study summary	Treatment type	IVIG/Plasma exchange dose	Results	Pain improvement score	References
FGFR3	2 (50% Female)	Two cases of young patients with progressive painful iSFN	IVIG	N/A	Dramatic improvement in Pain and numbness	N/A	Dave and Smith, 2018
FGFR3 and TS-HDS	40 (90% Females)	Retrospective analysis on patients with iSFN and positive for FGFR3 (27%) and TS-HDS (77%), or both (5%)	(*n* = 8) IVIG only + (*n* = 4) IVIG and Corticosteroids	2 g/kg/month loading dose followed by 1 g/kg/month	8 patients experienced a 44% reduction in pain and improved IENFD	−5.1 UENS −3.3 VAS (*P* = 0.02)	Zeidman and Kubicki, 2021
	17 (50% Females)	A double-blind placebo-controlled study with 17 patients positive for FGFR3 and/or TS-HDS	IVIG + Normal Saline (Placebo)	2 g/kg in 2 days loading dose followed by 1 g/kg/3 weeks	No significant change in IENFD and pain scores.	−1.8 ± 3.9 (IVIG) Vs. −3.0 ± 5.8 (Placebo) (*p* = 0.59) UENS	Gibbons et al., 2023
	1 (Female)	3 patients case series diagnosed with iSFN and high titers of FGFR3 and TS-HDS	IVIG	N/A	Improvement reported in the one and only patient treated with IVIG.	N/A	Bayraktutar et al., 2022
FGFR3 and TS-HDS and PLXND1	54 (74% Females)	A retrospective study on iSFN patients found 44% expressing autoantibodies of TS-HDS (62.5%), FGFR3 (29.2%), and PLXND1 (20.8%). 6 received treatment and had ENFD measurements taken.	(*n* = 6) IVIG + 3 patients received either Fremanezumab/ acetaminophen/ methylprednisolone	2 g/kg/month loading dose followed by 1 g/kg/month	Overall improvement in pain scores, but not significant due to the small sample size (5–6). 297% increase in the mean of IENFD. Higher titers of TS-HDS correlated with higher pain scores.	–13.4 VAS –20.8 UENS + 7.62 SFN-RODS –14.4 SFN-SIQ	Zeidman et al., 2022
TS-HDS	17 (71% Females)	patients associated with TS-HDS IgM autoantibodies, including 2 with FGFR3 autoantibodies	Plasma Exchange	3–5 procedures/2–3 weeks followed by one procedure/3–4 weeks	Pain in the upper (29%) and lower (41%) extremities improved in 71% of participants	NA	Olsen et al., 2022

VAS, Visual Analogue [pain] Score; UENS, Utah Early Neuropathy Score; SFN-RODS, SFN Rasch built overall disability scale; SFN-SIQ, SFN symptom inventory questionnaire.

In this review, we will discuss conflicting clinical data as well as preclinical studies pertaining to the presence of autoantibodies in SFN, and the idea that these are not just biomarkers but pathogenic, directly contributing to symptomology and thus providing insight into the cellular and molecular mechanisms relating to SFN.

## 2. Clinical evidence for a role of autoantibodies in SFN

Over the past two decades, a growing number of case reports and larger cohort studies have explored the application of immunotherapy as a treatment for SFN and in doing so the role of the immune system in SFN pathology. For example, early cases reports on a handful of patients highlighted the effectiveness of corticosteroid treatment as well as IVIG in SFN patients, particularly in reducing pain which would reoccur following termination of treatment (Dabby et al., 2006; Souayah et al., 2008). Another case series of 12 Sjögren’s syndrome associated SFN patients demonstrated that treatment with IVIG and subcutaneous immunoglobulin resulted in a significant reduction in pain intensity, as evidenced by a notable decrease in Visual Analogue Scale (VAS) scores, with 66% of the patients achieving complete resolution of symptoms (Pindi Sala et al., 2020). Recent development of B cell specific therapies such as rituximab provide more direct support for antibody involvement and in one case report has been shown to completely resolve neuropathic pain in a SFN patient with Sjogren’s syndrome (Venkatesh and Muley, 2022). More recent studies have investigated larger cohorts, allowing for a more comprehensive analysis of immunotherapy efficacy. A large retrospective study on 143 subjects with Sarcoidosis-associated SFN reported a favorable response in overall symptoms in 76% of patients treated with IVIG (Tavee et al., 2017). IVIG was also successful in decreasing pain scores by 50% after 6 months of treatment for Sjögren’s Syndrome-associated SFN (Gaillet et al., 2019). Moreover, a retrospective study of 55 autoimmune associated SFN patients showed that IVIG resulted in the average pain dropping significantly after 3 months of treatment (Liu et al., 2018). There is also evidence that autoantibodies contribute to iSFN. For example, plasma exchange therapy, successfully used for the treatment of many antibody-mediated neurological conditions, showed a 71% improvement in disease progression and symptoms in 17 patients suffering from pain in the upper (29%) and lower (41%) extremities (Olsen et al., 2022).

However, such studies are limited by their small numbers and retrospective analysis. In Geerts et al. (2021), a placebo-controlled, double-blinded, randomized trial was the first of its kind to test the effectiveness of IVIG on 60 patients diagnosed with painful length-dependent iSFN, with the primary outcome measuring pain reduction. The investigators reported a non-significant reduction in pain scores of 30% in the placebo group compared to 40% in the IVIG-treated group, arguing against the effectiveness of this treatment and hence dysimmunity in SFN pathology. It is of note however, that this study focused only on length dependent iSFN cases and therefore excluded SFN cases associated with autoimmune conditions as well as those with non-length dependent symptoms. This is of significance since one might suspect IVIG treatment to be most effective in such cases given previous studies had shown positive findings in SFN cases associated with autoimmunity (Liu et al., 2018; Gaillet et al., 2019). Therefore, while the study provides strong evidence of IVIG inefficacy as a treatment for iSFN, one cannot extrapolate this for all SFN patients, particularly those with overt or even more subtle dysimmunity.

## 3. Identification of autoantibodies in SFN patients and their pathogenicity

Although studies on the efficacy of IVIG for the treatment of SFN are conflicting, they do suggest that antibodies can contribute to disease. In line with this, autoantibodies have been identified (Pestronk et al., 2012; Antoine et al., 2015; Fujii et al., 2018; Chan et al., 2022), and it is important to understand their role in disease and symptom development.

Trisulfated heparin disaccharide (TS-HDS) is a cell-surface protein expressed by peripheral nerves. It can bind extracellular proteins and is implicated in specific functions such as cell growth and angiogenesis (Malik et al., 2019). Serum immunoglobulin M (IgM) binding to TS-HDS was first identified in a subset of patients with painful, distal axonal neuropathies (Pestronk et al., 2003) and has more recently been linked with SFN. For example, autoantibodies against TS-HDS (TS-HDS-Abs) have been found in patients with iSFN in a study by Levine et al. (2020), with more than 1/3 of patients having TS-HDS-Abs. Acute onset of disease is a hallmark of antibody-mediated conditions and > 90 percent of patients with acute-onset SFN were identified with TS-HDS-Abs which were associated with non-length dependent IENFD pathological findings (Levine et al., 2020). These findings are consistent with previous reports showing an increased frequency of pain in the upper extremities of patients with TS-HDS-Abs (Pestronk et al., 2012). It has been estimated that up to 43% of 40 iSFN patients have TS-HDS-Abs with severe neuropathic pain and non-length dependent loss of IENFD (Zeidman and Kubicki, 2021). As well as sensory abnormalities, autonomic dysfunction is also associated with TS-HDS-Abs with 28% of 322 SFN patients displaying dysautonomia (Trevino and Novak, 2021). IVIG treatment of TS-HDS-Ab positive SFN patients can improve symptoms including a significant reduction in the VAS pain scores and improvement in IENFD (see Table 1; Zeidman and Kubicki, 2021). Furthermore, 70% of TS-HDS-Ab positive patients reported either clinical improvement or slowing of their disease progression following therapeutic plasma exchange (Olsen et al., 2022). Although studies have not yet directly tested the pathogenicity of TS-HDS-Abs from SFN patients, muscle and nerve biopsies from sensorimotor polyneuropathy patients showed sensory axon loss with IgM and C5b9 complement deposition (Pestronk et al., 2012), suggesting complement activation as a possible mechanism.

Fibroblast Growth Factor Receptor 3 (FGFR3) is a cell surface protein belonging to the tyrosine kinase receptor family, and is linked to a variety of functions, including nerve regeneration, axon development, and damage-induced cell death signaling (Trevino and Novak, 2021). Interestingly FGFR3 expression is found on both small and large rat sensory neurons, as well as satellite glial cells (Antoine et al., 2015; Tholance et al., 2021). FGFR3-Abs were first identified in subgroup of sensory neuropathy patients and later confirmed in a smaller cohort of patients which included SFN cases (Antoine et al., 2015; Samara et al., 2018). The presence of FGFR-Abs has been linked specifically with SFN, confirmed in 15% of 155 iSFN patients who displayed non–length-dependent nerve pathology (Levine et al., 2020). Moreover, in a study on 322 SFN patients, 17% had FGFR3-Abs and were associated with a high neuropathy symptom score (including features such as aching pain, allodynia, burning pain, and prickling sensation) and autonomic symptoms (Trevino and Novak, 2021). The identification of these FGFR3-Abs has allowed specific targeting of positive cases and a number of studies have shown that IVIG treatment can reduce pain and improve pathology in these cases suggestive of pathogenicity (Dave and Smith, 2018; Zeidman and Kubicki, 2021; Bayraktutar et al., 2022). Epitope mapping has shown that patient antibodies can target functionally important regions of FGFR3 (Tholance et al., 2021), although these were mainly intracellular making their relevance to pathogenesis *in vivo* difficult to interpret. A recent double-blind placebo-controlled pilot study looking specifically at TS-HDS-Ab and FGFR3-Abs failed to detect a benefit of IVIG treatment on IENFD (see Table 1), although was inconclusive on pain outcomes due to the small number of participants (Gibbons et al., 2023), meaning the pathogenicity of FGFR3 and TS-HDS-Abs remains unclear.

Plexin D1 is another antibody target identified in SFN (Fujii et al., 2021). Plexin D1, is a large transmembrane glycoprotein that belongs to the plexin family, a group of factors involved in signal transduction and in axon guidance (Burk et al., 2017). Plexin D1-Abs, which are predominantly of the IgG2 subclass, were initially detected in patients with neuropathic pain associated with immune meditated conditions via their binding to mouse DRG sections and subsequent mass spectrometry analysis of immunoprecipitated samples (Fujii et al., 2018). Through the development of an ELISA assay, SFN patients were screened with around 13% positive for plexin D1-Abs (Fujii et al., 2021). A subsequent study has confirmed the presence of this antibody in separate patient cohorts and treatment with immunotherapy can reduce pain in these patients (see Table 1; Zeidman et al., 2022). In line with its known expression profile, patient antibodies can bind C-fiber nociceptors on mouse tissue sections, as well as nerve fibers in the skin and when patient IgG is applied to mouse DRG *in vitro* (in the absence of complement), they have cytotoxic effects (Fujii et al., 2018). Pathogenicity in terms of pain has been directly tested with passive transfer of patient IgG causing significant, but transient increases in both mechanical and thermal sensitivity (Figure 1) compared to healthy control IgG, 24 h after treatment (Fujii et al., 2021). Increased pain sensitivity in patient IgG treated mice was associated with pERK activation (Figure 1), perhaps through blocking plexin-D1s known ability to antagonize this pathway (Üçeyler et al., 2013), suggesting plexin D1-Abs are pathogenic in terms of pain in SFN patients through direct modulation of nociceptor physiology (Fujii et al., 2021).

A similar approach has been used to study IgG from patients with an acute SFN variant of Guillain–Barré syndrome (GBS) responsive to IVIG (Yuki et al., 2018). Patient IgG strongly bound small neurons in mouse DRG sections but not myelinated fibers from sciatic nerve. Transfer of patient IgG but not healthy control resulted in a transient increase in thermal pain responses in mice, suggesting the existence of pathogenic autoantibodies in SFN capable of directly targeting small fibers (Yuki et al., 2018). However, a target antigen was not identified. In addition, SFN is evident in FMS with around 40% predicted to have SFN and evidence indicates that FMS may have an autoimmune basis (Üçeyler et al., 2013; Goebel et al., 2021). Passive transfer of IgG from FMS patients to mice cause pain hypersensitivity and loss of intraepidermal innervation (Goebel et al., 2021).

The list of autoantibodies associated with SFN has been recently expanded. Using a high-throughput protein array technology, 9 novel autoantibodies have been identified including interferon-induced GTP-binding protein Mx1 (MX1), drebrin-like protein (DBNL), and cytokeratin 8 (KRT8), which were all associated with iSFN (Chan et al., 2022). Although pathogenicity has not yet been tested, the authors suggest a possible interaction between MX1 and TRPC6 which is expressed in mouse DRG and been suggested to play a role in neuropathic pain (Lussier et al., 2005; Elg et al., 2007). Interestingly subgroup analysis into iSFN and sSFN found antibodies against MX1 present at higher levels in iSFN, suggesting that if not pathogenic, MX1 may be used as a potential marker to differentiate between the two conditions (Chan et al., 2022). Interestingly autoantibodies targeting CASPR2 and LGI1, while associated with a range of neurological conditions pertaining to both the Peripheral Nervous System (PNS) and Central Nervous System (CNS), are associated with immunoresponsive neuropathic pain which is predominantly length dependent. As well as neuropathic pain, loss of IENFD have been described in these patients which can occur without CNS or large fiber involvement (Ramanathan et al., 2021), suggesting these antibodies are potentially pathogenic in SFN.

## 4. Discussion

It is well established that autoantibodies are a mechanism to cause peripheral neuropathy. For example, in inflammatory neuropathies such as GBS and Chronic Inflammatory Demyelinating Polyneuropathy (CIDP), antibodies targeting antigens within the PNS are known to drive pathology through mechanisms including macrophage or complement activation, as well as target disruption (Querol et al., 2023). Furthermore, autoantibodies can directly impact sensory neuron physiology by disrupting ion channel function (Dawes et al., 2018). Here we consider the role of autoantibodies in SFN.

One piece of evidence, in support of autoantibody involvement is the positive response of patients to therapies such as IVIG, plasma exchange or specific B cell targeting with rituximab (Tavee et al., 2017; Liu et al., 2018; Gaillet et al., 2019; Pindi Sala et al., 2020; Olsen et al., 2022; Venkatesh and Muley, 2022). IVIG represents the most commonly used approach, but its effectiveness has been brought into question following a lack of efficacy in a double-blind randomized placebo-controlled trial on painful iSFN patients (Geerts et al., 2021). This trial is important and overcomes the limitations of previous studies. For example, the placebo effect can be responsible for up to 30% of pain reduction which is a key outcome measure for SFN. However, others in the field have raised important questions regarding dosing strategy, exclusion rates and inclusion criteria and rightly called for caution in not extrapolating these findings to all of SFN (Lewis and Galetta, 2022). SFN treatment for known conditions can be targeted toward the underlying causes (Finsterer and Scorza, 2022). This is of course not the case in iSFN, which likely represents a heterogenous group of patients, although these may have similar, but less overt causes, as secondary cases. For example, the identification of autoantibodies in iSFN (Pestronk et al., 2012; Antoine et al., 2015; Fujii et al., 2021; Chan et al., 2022) suggests the presence of subtle dysimmunity in a subgroup of patients. Therefore, while the Geerts et al. (2021) study shows IVIG had no significant impact on pain in iSFN patients, a blanket consensus should not be applied to all SFN patients which may include subgroups of iSFN as well as those associated with autoimmune conditions, where, unsurprisingly, IVIG has been most successful (Liu et al., 2018).

It is clear that autoantibodies exist in patients with SFN, but one question is whether these are purely biomarkers or if they play a more direct role in pathogenesis. A recent proteomic study identified a number of new autoantibodies (Chan et al., 2022). However, many of these target intracellular proteins and therefore *in vivo* such antibodies would not have access to their target antigen and are therefore unlikely to drive pathology. Despite this, their existence implies dysimmunity and such antibodies (and their targets) could represent important diagnostic biomarkers. For example, autoantibodies were not present in all SFN patients and there was overlap between iSFN and sSFN cases. Therefore such antibodies could be useful in subgrouping patients and informing appropriate treatment strategies (Chan et al., 2022). TS-HDS and FGFR3-Abs have also been identified. While direct studies of their pathogenicity have not yet been performed, a recent placebo-controlled pilot study focusing on these patients showed a lack of IVIG efficacy (Gibbons et al., 2023). While this study was small and underpowered, particularly for pain outcomes, it makes the relevance of these antibodies to pathology less certain, although seropositivity could still be useful in diversifying patients (see Table 1; Zeidman et al., 2022). An alternative idea is that certain antibodies in SFN are pathogenic. This would not only help treatment, but where targets are identified give crucial insight into the molecular mechanisms underlying SFN which could be applicable to neuropathy in general and predominant symptoms such as neuropathic pain. Patient response to immunotherapy, appropriate binding of antibodies to relevant cells/tissue, *in vitro* assessment and passive transfer of symptoms in animal models are all key to confirming pathogenicity. Importantly, such studies have been carried out with patient IgG (Figure 1) and confirm the existence of pathogenic antibodies in SFN (Yuki et al., 2018; Fujii et al., 2021). It will be of great value to perform such studies on other known SFN autoantibodies (e.g., TS-HDS, FGFR3, MX1) so that their role in SFN pathology can be determined. One possibility in terms of pathogenicity is that these antibodies, rather than solely causing neuropathy, could drive neuropathic pain more directly by altering ion channel function in sensory neurons. For example, Plexin-D1-Abs induce pERK expression in sensory neurons. As well as being an activation marker, increased pERK is known to alter ion channel activity and increase the sensitivity of primary sensory neurons (Zhuang et al., 2004; Stamboulian et al., 2010). Furthermore, another autoantibody target MX1 regulates the activity of TRPC6. Increased calcium influx via TRPC6, enhances sensory neuron depolarization leading to increased pain hypersensitivity (Lewis and Galetta, 2022; Venkatesh and Muley, 2022). While further studies are of course needed to develop a clearer picture of how SFN antibodies might directly impact sensory neuron physiology, studies do support the idea that autoantibodies have a causal role in SFN pathology.

Another important question is whether additional, as yet unidentified pathogenic autoantibodies exist in SFN. The study by Chan et al. (2022) supports this notion, successfully using a protein array of 1,600 targets to uncover novel autoantibodies. Targets were selected based on involvement in the immune system, while this is of course logical, it is the small fibers which are affected in SFN and therefore important antigens may well have been missed. Furthermore, the 1,600 proteins assayed represent only a snapshot of known proteins, allowing for the prospect that additional autoantibodies could still be identified. In agreement with this, in the study by Yuki et al. (2018) where SFN patient IgG bound sensory neurons and caused pain hypersensitivity in mice (Figure 1), the target was not identified demonstrating that additional unknown antigens expressed on sensory neurons that are targeted in SFN are still yet to be uncovered. It is of note that in this study and that by Fujii et al. (2021), where pathogenicity was confirmed, patients were identified based on sensory neuron IgG binding (Figure 1), suggesting this as a key factor in autoantibodies having a more direct impact on pathology. These studies used tissue sections to identify patients with antibodies targeting sensory neurons. While this method has its advantages, it will expose antibodies to intracellular targets unlikely to convey pathogenicity. Another approach is to use live cultured sensory neurons, similar to that recently applied in GBS (Davies et al., 2022). This has the benefit of exposing patient IgG only to extracellular epitopes (in their physiological confirmation) that would be accessible *in vivo*, thus screening for putative pathogenic antibodies which are sensory neuron specific.

In summary, studies have unequivocally identified pathogenic autoantibodies in SFN, although it is unclear as to the exact prevalence of this as a mechanism in SFN as a whole. Future investigation looking for additional autoantibodies and the testing of their pathogenicity could provide a better understanding of the underlying molecular mechanisms in SFN and the development of neuropathic pain.

## Author contributions

JD: Conceptualization, Funding acquisition, Supervision, Writing–original draft, Writing–review and editing. OD: Writing–original draft, Writing–review and editing, and Visualization. AF: Writing–original draft, Writing–review and editing.
