# Corrigendum: A role for pathogenic autoantibodies in small fiber neuropathy?

**DOI:** 10.3389/fnmol.2023.1336871

**Published:** 2023-11-28

**Authors:** Omar Daifallah, Adham Farah, John M. Dawes

**Affiliations:** ^1^Departmemt of Zoology, King Saud University, Riyadh, Saudi Arabia; ^2^Nuffield Department of Clinical Neurosciences, University of Oxford, Oxford, United Kingdom

**Keywords:** autoantibodies, small fiber neuropathy (SFN), neuropathic pain (NeP), intravenous immunoglobulin (IVIG), passive transfer models, novel autoantibodies

In the published article, the funding statement was mistakenly not included in the publication. The Funding statement appears below.

